# The glycobiology of prostate cancer: an update

**DOI:** 10.1038/s41388-026-03858-x

**Published:** 2026-06-18

**Authors:** Kirsty Hodgson, Margarita Orozco-Moreno, Ziqian Peng, Libby Blencoe, Molly J. Sharp, Erin Smith, Grace Grimsley, David J. Elliott, Richard Beatson, Richard R. Drake, Jennifer Munkley

**Affiliations:** 1 Newcastle University Centre for Cancer, Newcastle University Institute of Biosciences, Newcastle, UK; 2 Department of Cell and Molecular Pharmacology, Medical University of South Carolina, Charleston, SC USA; 3 Centre for Inflammation and Tissue Repair, UCL Respiratory, Division of Medicine, University College London (UCL), London, UK

**Keywords:** Prostate cancer, Glycobiology

## Abstract

Prostate cancer is a common cancer in males and there is an urgent unmet clinical need to improve early diagnosis and identify new treatments for advanced disease. Despite huge progress in understanding changes to the genome and proteome in prostate cancer, there is a relative delay in revealing the full aspects of the prostate cancer glycome and glycoproteome. Glycobiology has been fundamental in recent discoveries in the medical field, including translational cancer research. Glycans functionally contribute to the cancer hallmarks and serve as important diagnostic biomarkers and targets for therapeutic intervention. Changes to glycans are common in prostate cancer and include increased branching of complex *N-*glycans, changes in sialylation, increased fucosylation, altered PSA glycosylation and the expression of truncated *O-*glycans. This review discusses the role of glycans in fundamental mechanisms controlling prostate cancer growth, metastasis and immune evasion. Emphasis is placed on discoveries made during the last decade, including new insights provided by *N-*glycan imaging mass spectrometry (IMS) profiling of prostate cancer tissues, new discoveries into the role of aberrant glycosylation in prostate tumour biology, as well as recent studies investigating glycans, glycosyltransferase enzymes and glycan binding proteins as therapeutic targets.

## Introduction

Prostate cancer is a common cancer in males and globally causes more than 350,000 cancer-related deaths every year [1]. When patients are diagnosed with early-stage (organ-confined prostate cancer), the 5-year survival rate is 95%, but this is reduced to only 50% for men diagnosed with advanced-stage or metastatic disease [2]. Guidelines highlight the importance of detecting prostate cancer while it is still locally confined and potentially curable [2]. However, unfortunately, there is a significant and rising trend of advanced-stage diagnoses [3]. While androgens are required for normal prostate function, in prostate cancer, the androgen receptor (AR) signalling axis is hijacked to promote the development and progression of prostate cancer [4]. Androgen blockade using drugs that prevent the production of androgens and/or block AR action is the cornerstone treatment for advanced prostate cancer and new AR-targeted therapies, including enzalutamide, abiraterone [5] and upfront combination therapies [6, 7] have improved patient outcomes. However, resistance to hormonal therapy, known as castrate resistant prostate cancer (CRPC) [8], is almost inevitable and there is a critical need to develop new therapeutic options to eradicate tumours. An understanding of the molecular features associated with prostate cancer progression and resistance to therapy is crucial to identify new diagnostic biomarkers and targets for therapy.

Glycosylation is a vital biological process that is crucial for processes like cell recognition, protein folding, cell signalling and immunity, with errors in glycosylation linked to numerous diseases [9–12]. Glycans are carbohydrate molecules composed of monosaccharides (sugar units) linked together by chemical bonds. Alongside nucleic acids, proteins and lipids, glycans are one of the four major macromolecules of life and play fundamental roles in a wide variety of biological processes [13, 14]. The glycome, which includes all glycans synthesised by cells, is potentially as important to our understanding of life as the genetic code. Historically, our knowledge of these major macromolecules has lagged due to the structural complexity of glycans and the lack of tools to investigate/analyse glycosylation [15]. The biosynthesis of glycans is determined by the action of glycosyltransferases (writers) and glycosidases (erasers) that mediate the assembly, processing and turnover of glycans. Glycan recognition is performed by lectins (readers), which are proteins that bind to specific glycans [16]. There are two main types of protein glycosylation, *N*-glycosylation and *O*-glycosylation. *N*-glycosylation starts in the endoplasmic reticulum (ER), where a common oligosaccharide precursor (Glc3Man9GlcNAc2) is attached to asparagine residues of proteins, before further processing and maturation into various structures (complex, hybrid, or high-mannose) in the Golgi apparatus [17]. *O*-glycosylation primarily occurs in the Golgi apparatus, where *N*-acetylgalactosamine (GalNAc) is added to serine or threonine residues on proteins, followed by the sequential addition of further monosaccharides [18].

**Fig. 1:**
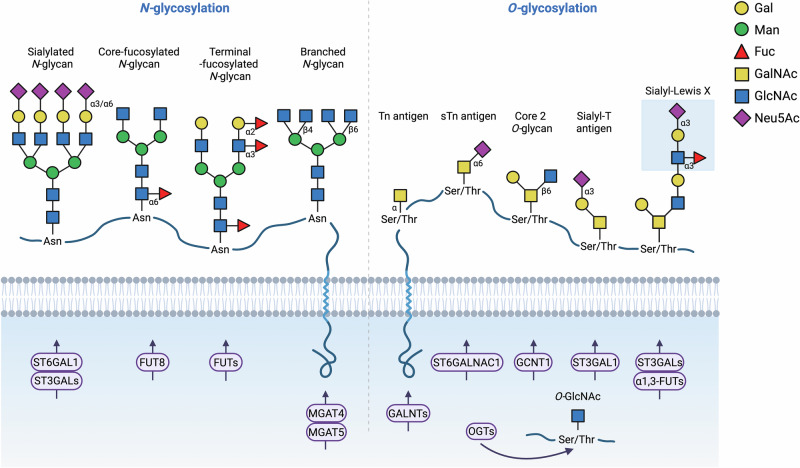
Schematic representation of important glycan structures that are commonly altered in prostate cancer. Common changes to glycans in prostate tumours include increased branching of complex *N*-glycans, changes in sialylation, increased fucosylation and alterations to *O*-glycans.

Aberrant glycosylation is common in cancer cells and is functionally linked to all of the cancer hallmarks [12]. In prostate cancer, common changes to glycans include increased branching of complex *N-*glycans, changes in sialylation, increased fucosylation, changes to PSA glycosylation and alterations to *O-*glycans [19, 20] (Fig. 1). In 2016, our group published a review about glycosylation in prostate tumours, which covered the potential of glycans to improve the diagnosis and treatment of prostate cancer [20]. Now, ten years later, this current review highlights advances in this area in the last decade and how this potential is being realised. In particular, we emphasise new insights provided by *N-*glycan imaging mass spectrometry profiling of prostate cancer tissues, as well as recent studies exploring the role of glycans in prostate cancer progression and their potential as diagnostic biomarkers and therapeutic targets.

## *N*-glycan branching

An important technological advance has been the ability to profile *N*-glycans in prostate tumours. *N*-glycosylation leads to a wide variety of structures with significant changes in the abundance of specific *N-*glycans known to occur in cancer [10]. Numerous studies have reported an increase in multi-antennary *N*-glycans on serum glycoproteins both in prostate cancer and in CRPC [21–23]. These branched structures are synthesised by dedicated glycosyltransferases, including the *N*-acetylglucosaminyl transferase enzymes MGAT3, MGAT4, MGAT5 and MGAT5B. However, the mechanisms regulating the formation of β-1,6-GlcNAc branching are yet to be fully elucidated [24, 25]. MGAT5 has been shown to play a role in metastatic prostate cancer [26] and the ER degradation enhancer mannosidase α-like 3 (EDEM3), which promotes mannose trimming of *N-*glycans, is upregulated in prostate tumours and is linked to radio-resistance [27]. Furthermore, metabolic reprogramming (the Warburg effect) becomes more pronounced in advanced prostate cancer [28] and may be a general mechanism to promote *N-*glycan branching [29]. In colorectal cancer, the intratumoral T-cell glycome is altered with substantial changes in branched *N*-glycans reported [30]. A recent study showed that reprogramming of CD8+ T cell branched *N*-glycosylation, using MGAT5 knockout anti-CD19-CAR T cells, limits exhaustion, enhances cytotoxicity and promotes tumour killing [30]. Although changes to the intratumoral T-cell glycome in prostate cancer have yet to be reported, investigating the potential role of MGAT5-mediated branched *N*-glycans in regulating CD8+ T-cell function represents a promising area to explore.

**Fig. 2:**
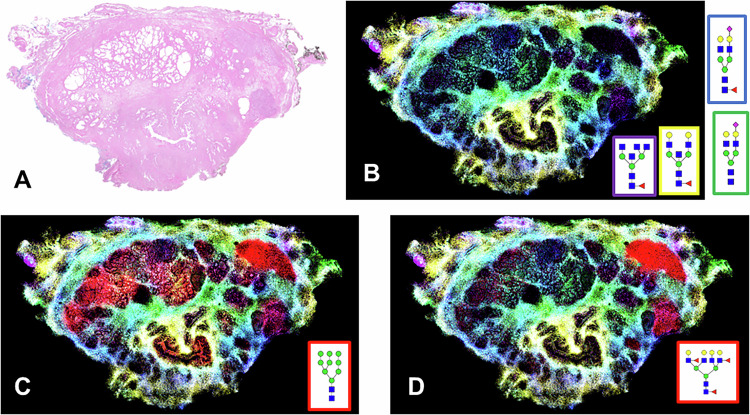
Example *N*-glycan MSI image of a typical prostate cancer tissue with representative *N*-glycan distributions. FFPE prostate cancer tissue was processed for *N*-glycan MALDI-MSI as described [33]. **A** H&E stain. **B** Four stroma and inflammation *N*-glycan distributions. The spatial colours correspond to the border colour for each glycan structure. The same four glycans are shown in (**C**) with a high mannose glycan included in red and **D** with a representative tetra-antennary tumour *N*-glycan in red.

The development of *N-*glycan mass spectrometry imaging (MSI) by the Drake lab [31, 32] has allowed glycans in pure tumour regions to be directly profiled on formalin-fixed, paraffin-embedded (FFPE) tissue specimens from patients. This has led to pivotal advances in the field of glycobiology [33, 34], including effective modelling of glycosylation changes throughout the prostate cancer evolution. In Fig. 2, an example *N*-glycan MSI image of a typical prostate cancer tissue is shown with representative *N*-glycan distributions. Using *N-*glycan MSI, Butler et al. [35] profiled *N-*glycans in tumour regions from 131 prostate cancer patients representing the disease spectrum from early hormone-sensitive to late therapy-resistant tumours. Although early-stage prostate cancers had high interpatient variability, with few glycans demonstrating consistent changes across patients, prostate tumours treated with hormonal therapy had increased levels of complex biantennary structures with decreased levels of tri- and tetra-antennary glycans and in prostate tumours resistant to hormonal therapy, tri- and tetra-antennary glycans were aberrantly upregulated [35]. In further studies, Hartig et al. utilised *N-*glycan MSI to monitor *N-*glycans in a novel outcome cohort of prostate cancer tissues, including primary prostatectomy samples categorised as ‘no evidence of disease’ or ‘metastasis’ based on more than 5 years of disease progression outcomes [36]. This analysis showed an increase in *N-*glycan complexity in metastatic prostate tissue, including multi-branched *N-*glycans containing sialic acid and multiple fucose and terminal GlcNAc structures [36]. Conversely, complex *N-*glycans were downregulated in patients with a rare aggressive form of neuroendocrine prostate cancer called small cell neuroendocrine carcinoma of the prostate [37]. This is potentially because neuroendocrine cells produce fewer glycoproteins than luminal cells [35]. Further studies will be needed to comprehensively profile *N-*glycan branching in other uncommon prostate cancer subtypes, including AR-negative neuroendocrine tumours, carcinoid, amphicrine and squamous carcinomas. The underlying mechanisms and functional consequences of *N-*glycan branching in prostate cancer pathology remain to be fully investigated, but likely include alterations to the binding of Galectins (proteins which bind to β-galactoside glycans on the cell surface and modulate the tumour microenvironment [38, 39]). These findings suggest increased *N-*glycan branching is closely linked to prostate cancer metastasis and the development of CRPC and identify highly branched *N-*glycans as potential therapeutic targets that could be exploited to benefit prostate cancer patients who have exhausted currently available treatment options.

## Terminal fucosylation

A common cancer-associated change in glycosylation is aberrant fucosylation [10] and altered fucosylation has long been associated with aggressive prostate cancer [40]. A family of 13 fucosyltransferase (FUT) enzymes catalyse fucosylation, where fucose residues are attached to glycans. Fucosylation can be divided into either terminal or core fucosylation [41–44]. Fucosyltransferase −3, −6 and −7 (FUT3, FUT6 and FUT7) are elevated in bone and liver metastatic prostate cancer, correlate with the synthesis of E-selectin ligands on prostate cancer cells and are master regulators of prostate cancer cell trafficking to the bone marrow [45, 46]. Studies exploring the role of fucosylated *N-*glycans in cellular interactions between prostate-derived tumour cells and the metastatic tumour microenvironment are ongoing.

Recently, we investigated the effects of global metabolic inhibitors of fucosylation in the context of prostate cancer progression [47]. Metabolic inhibition of fucosylation was shown to decrease the growth of prostate cancer cells in vitro and to downregulate the expression of oncogenic genes and proteins, highlighting the importance of fucosylation in the trajectory of prostate cancer progression [47]. *N*-glycan MSI studies showed hyper-fucosylated *N-*glycans are enriched in prostate adenocarcinomas that progress to metastatic disease and in neuroendocrine prostate cancer and an analysis of patient-derived xenografts revealed this fucosylation signature is highest in prostate-derived tumours metastasised and growing in the liver [48]. Terminal fucosylation is known to impact the synthesis of selectin ligands that regulate interactions between cells and the endothelium to control processes such as tumour cell migration and lymphocyte homing [49, 50].

## Core fucosylation

Altered core fucosylation of *N-*glycans is a common change in tumour glycan patterns contributing to cancer growth, metastasis and immune evasion [42, 51–53]. In prostate cancer, an increase in core-fucosylated *N-*glycans has long been associated with advanced disease [54–56]. Core fucosylated PSA glycoforms in patient serum and urine samples have been investigated as diagnostic biomarkers [55, 57–63], while serum fucosylated haptoglobin also holds potential to predict high-grade disease [64]. Newly developed MALDI-MSI technologies have been applied to investigate whether glycan signatures in organ-confined prostate cancers can be used to assess the potential risk of future disease recurrence. A recent study utilised *N*-glycan MSI to document *N*-glycome alterations in tumours and revealed that while total core fucosylation levels do not change in prostate cancer relative to normal prostate tissue, it is likely that the specific *N*-glycans being core fucosylated are the important factor [65]. In prostate cancers with high risk of progression, increased levels of core-fucosylated *N-*glycans alongside an overall increase in the abundance of *N-*glycan structures have been detected, suggesting these molecular features have potential as early predictors of high-risk disease and metastasis [36].

Core fucosylation is catalysed by the α1,6 fucosyltransferase 8 (FUT8) enzyme, which transfers fucose to the innermost GlcNAc of *N*-linked glycoproteins in α1,6 linkage [42, 51, 66]. FUT8 is the only FUT enzyme responsible for core fucosylation and as most other fucosyltransferases are functionally redundant, this makes FUT8 and core fucosylation a potential ‘Achilles heel’ for prostate cancer [67–71]. FUT8 plays an important role in cancer biology and can regulate the activity of growth receptors and adhesion molecules to increase cell proliferation, metastasis and immune evasion in many cancers [42]. In prostate cancer, studies suggest FUT8 is upregulated in high-grade tumours and is linked to the development of CRPC [54, 72, 73]. Recently, we monitored FUT8 levels in clinical samples across multiple patient cohorts and verified upregulation of FUT8 as a feature of aggressive prostate cancer [74]. Our study showed FUT8 underpins the synthesis of malignant core fucosylated *N-*glycans in prostate cancer cells and suggested FUT8 regulates prostate cancer growth and can be targeted using metabolic fucosylation inhibitors to suppress the growth of prostate tumours [74]. Mechanistically, FUT8 correlates with the expression of genes and signalling pathways linked to prostate cancer progression [74] and although the specific *N*-glycoproteins modified by FUT8 in prostate cancer are yet to be identified, it is clear that the role of FUT8 in cancer is multifaceted, likely involving the regulation of cell signalling receptors, cytokines and immune checkpoint molecules [75]. In triple-negative breast cancer, FUT8-mediated core glycosylation of the B7H3 immune checkpoint protein can inhibit T cell activity [76, 77]. Similarly, core fucosylation of the programmed cell death 1 (PD-1) can suppress the activity of cytotoxic T lymphocytes in lung cancer [78]. These findings suggest core fucosylation will likely also regulate the stability of immune checkpoint receptors in prostate tumour cells, although more research is needed in this area.

## Sialylated *N-*glycans

**Fig. 3:**
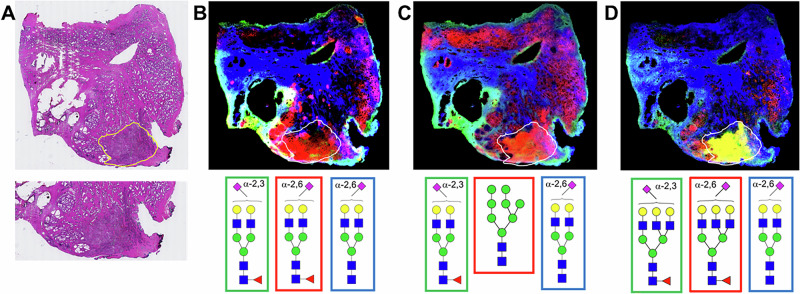
Example of *N*-glycan MSI image of a representative prostate cancer tissue showing stabilized α2,3 and α2,6 sialylated *N*-glycan distributions in stroma and tumour regions. FFPE prostate cancer tissue was processed for *N*-glycan amidation stabilization of sialic acid isomers prior to MALDI-MSI as described [88]. **A** H&E stain with tumour region highlighted in yellow. **B** Distribution of three bi-antennary singly sialylated *N*-glycan distributions. The spatial colours correspond to the border colour for each glycan structure. **C** The same stroma glycans (in blue and green) are included with a high mannose glycan (in red). **D** Two different tumour glycan distributions are detected based on α2,3 (green) or α2,6 (red) sialic acid linkages.

Sialylated *N*-glycans are often altered in cancer and their upregulation (hypersialylation) on the surface of cancer cells is closely linked to tumour progression, metastasis and patient survival. α2,3 and α2,6 sialylated *N-*glycans are the most abundant isomers of sialic acid detected in humans. Alterations to both α2,3 and α2,6 sialylated *N-*glycans are common in prostate cancer and this has important implications for tumour progression, metastasis and immune evasion [79]. Studies from over 30 years ago have reported the expression of Lewis antigens in prostate tumour tissues, indicating that Sialyl Lewis X (SLe^X^) and Sialyl Lewis Y (SLe^Y^) are linked to metastatic disease [80–84]. Furthermore, SLe^X^ has been associated with poor prognosis in patients undergoing hormonal therapy [84, 85]. *N*-glycan MSI studies have also identified larger branched α2,6 sialylated *N-*glycans in prostate tumour tissues [86]. New advances using a combination of double amidation isomer tagging and *N*-glycan MSI have advanced the identification of sialylated *N-*glycans in patient tissue samples [87, 88]. An N-glycan MSI example of a representative prostate cancer tissue showing stabilised α2,3 and α2,6 sialylated *N-*glycan distributions in stroma and tumour is shown in Fig. 3. MALDI-MSI high-resolution spatial analysis of α2,3 and α2,6 sialylated *N-*glycan isomers in 381 primary prostatectomy samples in tissue microarray format revealed significant differences in specific *N-*glycans in patients with Gleason score 6, 7[3 + 4] or 7[4 + 3] disease, including upregulation of α2,3 linked tetra-antennary *N-*glycans, downregulation of bi-antennary α2,3 and α2,6 *N*-linked structures and fluctuations in tri- and tetra-antennary glycans of both isomers with tumour advancement. [89]. Prostate-derived cancers growing in bone also contain both α2-6 and α2-3 linked sialic acids, with more tri-antennary sialylated *N*-glycans represented in bone metastatic tissue compared to primary prostate tumours and an increase in *N-*glycans with two or more α2-6 sialylated species [90]. We have also detected unique single α2-6 sialylated poly-lactosamine containing *N*-glycans in bone metastatic prostate tissues [90], which are linked to poor clinical outcomes in metastatic breast cancers [91]. Together, these findings indicate remodelling of sialylated *N-*glycans with prostate cancer progression, with larger α2-6 sialylated tri-antennary and tetra-antennary *N*-glycans at high levels in prostate tumours growing in bone.

ST6 β-galactoside α-2,6-sialyltransferase 1 (ST6GAL1) catalyses the addition of α2,6-linked sialic acids to terminal *N-*glycans to modify glycoproteins and/or glycolipids and plays a key role in multiple mechanisms intrinsic to tumour cell biology [92]. Studies have correlated the overexpression of sialyltransferase enzymes that underpin the biosynthesis of malignant glycans to cancer progression [93–98]. In prostate cancer, ST6GAL1 is upregulated in aggressive tumours and aberrant α2,6-linked sialylation is linked to disease progression, bone metastasis and therapy resistance [86, 90, 99, 100]. Mechanistically, the role of ST6GAL1 in aggressive prostate cancer is multi-faceted, involving the regulation of immunosuppressive sialoglycans, promotion of M2-like macrophages and modification of the bone pre-metastatic niche towards bone resorption to promote the vicious cycle [90]. Although canonically ST6GAL1 resides in the intracellular secretory apparatus and glycosylates nascent glycoproteins, ST6GAL1 is also released into the extracellular milieu and is present in blood samples from prostate cancer patients [86]. The impact on prostate cancer cell biology of this non-canonical extrinsic mechanism of ST6GAL1 was previously unknown. However, recent findings indicate ST6GAL1 can be released by cancer cells in small extracellular vesicles and can modulate cell surface sialylation on recipient cells [101].

## PSA, PAP and PSMA glycosylation

Glycans provide the potential to augment current liquid biopsies for prostate cancer. Physiologically, the prostate is a secretory gland that produces thousands of glycoproteins present in prostatic fluids. One of the more abundant glycoproteins specifically produced by the prostate gland is prostate-specific antigen (PSA), a serine protease that liquefies semen [102]. Normally, only a minute amount of PSA leaks into the circulating blood, but in prostate cancer patients, there is a disruption of the prostatic epithelium, allowing increased release of PSA into the bloodstream. The PSA blood test can often identify patients with prostate cancer [103]. However, PSA levels in blood circulation can also increase in patients with prostatitis, benign prostatic hyperplasia (BPH) and in men with clinically insignificant disease [102], meaning PSA lacks the required sensitivity and specificity to be used as an accurate screening tool [104]. The diagnostic accuracy of PSA testing can be improved by detecting total PSA levels in combination with other biomarkers or alternative PSA isoforms such as free PSA [105], but further refinements are needed to improve identification of clinically significant prostate cancer. PSA is a glycoprotein with a single *N*-glycosylation site at asparagine (Asn)-69 [20]. The most abundant PSA glycoform in healthy men is a complex type bi-antennary oligosaccharide [106], although a genetic polymorphism (SNP), with an allele frequency of 4%, has been reported to lead to an additional glycosylation site [107].

Advances in prostate glycobiology provide the means to potentially enhance the efficacy of the current PSA test. Numerous studies have sought to detect different PSA-glycoforms in blood or urine samples to provide diagnostic and prognostic information [61, 108, 109]. Findings suggest that serum PSA from patients with prostate cancer may have increased levels of fucosylation [110], as well as increased α2,3-linked sialic acid [111]. More recent studies suggest serum core fucosylated PSA holds value for the detection of high-risk prostate cancer [59] and that altered PSA glycosylation could be used to identify patients with CRPC [112]. It has also recently been reported that PSA glycoforms are altered in situ in prostate cancer tissue compared to adjacent benign prostate tissue [113]. A study published in 2024 showed that the detection of two free PSA glycoforms, recognised by WFL lectin and PHA-E lectin, which bind the *N*-acetyl sugar residues GalNAc, LacdiNAc and bisecting GlcNAc, outperforms total PSA and PSA-based Prostate Health Index (PHI) tests and has significant clinical potential to detect prostate cancer [114]. While clinically, serum/plasma are routinely used for measurement of PSA protein levels, as PSA is detected at low levels (nanogram/mL) in blood, this often makes analysis of circulating PSA glycoforms challenging. PSA levels are much higher in seminal fluids (milligram/mL), expressed prostatic secretions (EPS) and urine (microgram/mL) and numerous studies have focused on evaluating PSA glycoforms in these fluids (reviewed in [108]). Together, these findings point to the future development of a glycosylation-based PSA test to improve prostate cancer diagnosis. However, it should be noted that in PSA glycoform studies with high sensitivity values, specificity values are often low and it has been suggested that it will be necessary to monitor multiple glycan structural changes to improve diagnostic performance. In addition, many PSA glycoform studies do not fully represent the patient groups with the highest clinical need for improved diagnosis and further studies using larger, more clinically relevant patient cohorts are needed to translate these findings into patient benefit.

Similar to PSA, Prostatic acid phosphatase (PAP) is a glycoprotein mainly synthesised in prostate epithelial cells [115]. Prior to PSA, PAP was investigated as a marker for prostate cancer detection, but was shown to exhibit non-specific expression in multiple organs [116]. PAP contains three *N*-glycosylation sites and the glycosylation of PAP is critical for its stability [117]. Alterations to PAP glycosylation have been correlated to prostate cancer in numerous studies [118], with increased fucosylation and decreased sialylation detected in cancerous prostate tissue compared to healthy control tissues [119]. In 2023, Wang et al. established an in-depth glycoproteomic assay for characterisation of PAP in urine, which importantly can distinguish α2,3 from α2,6-linked sialylation and enables in-depth characterisation of the PAP *N*-glycome [118]. Although it has only been tested on a limited number of patient samples so far, the study provides an important foundation to verify previous discoveries and evaluate the diagnostic potential of PAP glycoforms in prostate cancer.

Prostate-Specific Membrane Antigen (PSMA) is a transmembrane protein expressed in prostatic tissues and is a diagnostic and possibly therapeutic target [120]. High expression of PSMA is associated with high-grade prostate tumours [121]. Clinically, PSMA is used to improve imaging in prostate cancer detection [122] and has been explored as a non-invasive biomarker [123, 124]. PSMA is heavily glycosylated, with 10 predicted glycosylation sites and glycans making up 30% of its molecular mass [125]. Recent findings suggest glycosylation of PSMA by MGAT5 is required for its expression and identify a potential link with tumour malignancy through activation of STAT3 [126]. However, relative to PSA, PSMA glycoforms have been understudied as diagnostic biomarkers [127]. Alterations to PSMA glycoforms include increased *N-*glycan branching [126] and changes to sialylation [128] and fucosylation [129]. Aberrantly glycosylated PSMA can be detected in patient urine and assays that detect PSMA glycosylation are beginning to show promise to improve prostate cancer diagnosis. For example, macrophage galactose-type lectin (MGL) conjugated nanoparticles can be used to detect a PSMA glycovariant to discriminate prostate cancer from benign conditions [130], although further studies using larger patient cohorts are needed to fully assess clinical performance.

## *O-*glycans

*O*-glycans represent one of the most abundant and diverse glycan types, found on ~80% of proteins travelling through the secretory pathway [131]. Mucin-type *O*-glycosylation occurs in the Golgi apparatus, where GalNAc is added to serine/threonine residues on proteins, followed by the step-wise addition of additional monosaccharides [18]. Historically, the *O-*glycome has remained incompletely characterised due to structural complexity, its dynamic nature and analytical challenges [15]. Unlike *N*-glycosylation, there is no pre-formed precursor for *O*-glycans and *O*-glycans are dynamic modifications that change rapidly in response to cellular conditions. Until recently, there was no identification of a single analogous endoglycosidase enzyme for *O*-glycans (like PNGaseF is used to release *N*-glycans from proteins). However, advances in glycoproteomics in recent years have led to emerging studies that provide insights into how mucin-type *O*-glycosylation is altered in cancer [132, 133]. Kawahara et al. utilised sensitive glycomics and glycoproteomics to map *O-*glycans in surgically removed prostate cancer tissues. This identified tumour grade-specific dynamic remodelling of the prostate cancer *O-*glycome, with a significant reduction in sialylated core-1 and increased sialylated core-2 structures during disease progression [134]. Correlations between the *O-*glycoproteome and prostate cancer progression were also identified, including a robust increase in site-specific core 2 type *O*-glycosylation of collagen VI in higher-grade tumours [134]. Studies suggest core 2 *O-*glycans are linked to natural killer cell immunity and prostate cancer metastasis [135]; however, the specific role of core 2 *O-*glycosylation of collagen VI in prostate cancer has not yet been investigated.

The aberrant expression of glycosyltransferase enzymes is a key mechanism underlying changes to *O-*glycans in cancer [136] and numerous *O*-glycosyltransferase enzymes have been implicated in prostate cancer progression, including GALNT7, ST6GALNAC1, GCNT1 and ST3GAL1. Upregulation of N-acetylgalactosaminyltransferase 7 (GALNT7), which is part of a family of 20 enzymes that initiate mucin-type *O*-linked glycosylation, is a feature of prostate cancer that modifies *O*-glycosylation and promotes tumour growth [137]. The levels of GALNT7 are significantly higher in prostate cancer tissues relative to normal prostate tissue and GALNT7 levels remain high in therapy-resistant prostate cancer [137]. Furthermore, GALNT7 is also upregulated in urine and blood samples from men with prostate cancer and is being explored as a diagnostic biomarker for the early detection of clinically significant disease (NCT06554587). Mechanistically, GALNT7 correlates with cell cycle and immune signalling pathways in prostate cancer cells, including the regulation of tumour suppressor FOXO1. A ‘bump and hole’ chemical reporter system demonstrated that GALNT7 modifies the secretome of prostate cancer cells and, using site-specific *O*-glycoproteomics, 34 glycopeptides have been identified as potential enzyme substrates [137]. GALNT7 is also linked to altered expression of the Thomsen-nouveau (Tn) antigen (GalNAcα1-Ser/Thr) in prostate cancer cells [137]. While Tn is normally modified in the Golgi apparatus into more complex *O*-glycans, in many cancers the biosynthetic pathway is truncated, leading to abnormal expression of truncated *O-*glycans on the cell surface [138]. Historical data estimated that 90% of prostate cancers express the Tn antigen; however, a more recent analysis of 77 prostate tumours using different antibodies indicated that only 4–26% of prostate cancers are Tn positive [139]. Mechanistically, the Tn antigen is linked to metastasis [140] and binding of Tn to the MGL receptor on macrophages and dendritic cells contributes to an immune suppressive microenvironment [141]. Although the specific functional role of Tn in prostate cancer remains to be investigated, studies from other types of cancer highlight the potential importance of Tn in prostate cancer biology and point to the need for further studies in this area.

A glycan structure called sialyl-Tn (sTn) antigen is detected on many cancers, where it is associated with disease progression and poor prognosis [138, 142–145]. The sTn antigen is formed by the enzyme ST6 N-acetylgalactosaminide alpha-2,6-sialyltransferase 1 (ST6GALNAC1) that catalyses the addition of a sialic acid residue onto the Tn antigen to form the sTn antigen (Neu5Acα2-6GalNAcα1-O-Ser/Thr), a terminal structure that cannot be further elongated [146–148]. ST6GALNAC1 is upregulated in prostate cancer and this correlates with the expression of sTn [149], which has been detected in up to half of high-grade prostate tumours [150, 151]. Levels of the sTn antigen in prostate cancer cells may increase under hypoxia [152] and in response to androgens, and have been linked to reduced cell adhesion and a more mesenchymal cell phenotype [149]. A comprehensive analysis of ST6GALNAC1 and sTn in prostate cancers representing the full clinical heterogeneity of the disease has not yet been reported, but will be required to assess the expression of sTn in different disease stages, metastasis and the development of therapy resistance. Advances over the last decade demonstrate that sTn can bind immune cells via specific lectins (including MGL and Siglecs) to inhibit Natural Killer (NK) cell cytotoxicity, dendritic cell maturation and T cell activation and promote immune suppression in the tumour microenvironment [153]. Although the specific role of sTn in prostate tumour immune suppression has not yet been investigated, our recent findings suggest Siglec-15 (which is known to engage sTn [154]) is expressed by macrophages in the prostate tumour immune microenvironment [155]. Multiple studies have identified Siglec-15 as a central glyco-immune checkpoint [156, 157], pointing to the need for further studies to investigate the potential role of the sTn-Siglec-15 axis in prostate cancer biology.

The *O*-glycosyltransferase Glucosaminyl N-acetyl transferase 1 (GCNT1), which catalyses the formation of core 2 branched *O-*glycans, has been widely studied in the context of prostate cancer. Several studies have demonstrated that GCNT1 is upregulated in prostate tumours and linked to recurrence after surgery and the spread of prostate cancer cells outside of the prostate gland [158–161]. Increased GCNT1 can enhance the growth of prostate tumours [160, 162] and correlates with higher levels of core 2 *O*-SLe^X^ on PSA, PAP and MUC1 proteins [158]. In line with previous findings, we recently showed GCNT1 can alter the glycome of prostate cancer cells to increase levels of core 2 *O-*glycans (including the SLe^X^ structure). Furthermore, we identified 60 potential substrates for GCNT1 in the prostate cancer secretome and showed that upregulation of GCNT1 in prostate cancer cells can impact oncogenic signalling pathways [160]. Notably, SLe^X^ is a Selectin ligand with a key role in metastasis [163] and has been linked to the promotion of prostate cancer metastasis by mediating prostate cancer cell binding to E-selectin [46].

ST3 beta-galactoside alpha-2,3-sialyltransferase 1 (ST3GAL1) catalyses the transfer of sialic acid in α-2,3 linkage to galactose-containing substrates [164]. Studies have investigated ST3GAL1 in breast cancer, where it is responsible for the aberrant sialylation of MUC1, which correlates with tumour grade and promotes tumour growth [164, 165]. In prostate cancer, ST3GAL1 is a suggested biomarker to predict prognosis and monitor disease progression [93] and is increased in CRPC [166]. Studies using prostate cancer cell lines showed that upregulation of ST3GAL1 blocks *O-*glycan elongation, leading to increased levels of the sialyl-T antigen and reduced apoptosis [167, 168]. ST3GAL1 is a key enzyme responsible for the synthesis of sialoglycans that can engage Siglec receptors on immune cells [169]. Recent findings show ST3GAL1 can modulate prostate tumour immune evasion through the synthesis of ligands for Siglec-7 and Siglec-9 and suggest the levels of ST3GAL1 in prostate tumours negatively correlate with androgen signalling [170]. Taken together, these studies highlight the critical importance of *O*-glycosyltransferase enzymes and *O-*glycans in prostate cancer pathology and suggest further studies investigating the prostate cancer *O-*glycome will ultimately lead to an improved molecular-level understanding of disease mechanisms.

## Glycan-mediated immunoregulatory networks in cancer

Changes in glycosylation in cancer have functional consequences on the immune system, with tumour-associated glycans establishing glyco-immunoregulatory networks to promote immune evasion mechanisms [171]. Cancer glycosylation plays a central role in tumour immune editing and contributes to self-associated molecular patterns [172], which are recognised by glycan-binding proteins (GBPs). GBPs, including sialic acid-binding immunoglobulin-like lectins (Siglecs) and Galectins, are expressed and secreted by a range of immune cells in the tumour microenvironment [173–177]. Siglecs are a family of GBPs that are expressed on immune cells and interact with sialoglycans. Humans have 14 Siglecs [178] and many Siglec receptors have immunotyrosine-based inhibitory (ITIM) or switch (ITSM) motifs and are inhibitory [179]. Siglec-sialoglycan interactions likely act as a form of ‘self’ recognition to prevent autoimmune responses [180]. However, many cancer cells upregulate sialoglycan ligands which can engage inhibitory Siglecs, leading to the modulation of immune cell activity within the tumour microenvironment, with relevance to T cells, NK cells, macrophages, dendritic cells and neutrophils [156, 181–186].

Galectins are a family of GBPs characterised by a conserved carbohydrate-binding domain that binds to β-galactosidase on other molecules [187]. Galectins have a wide range of biological functions and have well-established roles in cancer progression and metastasis. This is likely by modulating interactions between tumour cells and the surrounding tumour microenvironment [188, 189]. Galectins modulate immune cells by mediating extracellular clustering of receptor complexes and influencing intracellular signalling and apoptosis [190]. There are 12 galectins in humans and in cancer, galectins can influence T cell activity and induce tolerogenic dendritic cells and immune-suppressive macrophages to support tumour growth and immune escape [191–193]. With mounting evidence that GBPs regulate immune cell function in cancer, there has been a big push to develop therapeutic approaches to inhibit GBP-glycan interactions, which is explored in more detail in the context of prostate cancer below.

## Siglecs

Recent studies have identified a critical link between prostate cancer glycobiology and Siglecs, which, along with their sialoglycan ligands act as ‘glyco-immune checkpoints’ to facilitate tumour immune evasion [177, 194, 195]. Findings suggest sialoglycans with the capacity to engage Siglec receptors may be upregulated on the surface of prostate cancer cells [155, 170, 184, 196] and studies have functionally linked altered sialylation to prostate cancer growth, bone metastasis and anti-tumour immunity [86, 90, 170]. Siglec-7 and Siglec-9 correlate with poor prognosis in prostate cancer patients and are highly expressed by myeloid cells, including macrophages in prostate tumour tissues [170, 184]. Mechanistically, sialylated glycoproteins on prostate cancer cells can suppress immune cell killing by binding to Siglec-7 and Siglec-9, with CD59 identified as a candidate ligand for Siglec-9 [184]. Our recent findings reveal a significant upregulation of Siglec-engaging immunosuppressive sialoglycans in prostate tumours growing in bone, including ligands for Siglec-3, -7 and -9. Furthermore, Siglec receptors are expressed by immune cells in the bone metastatic prostate tumour immune microenvironment and sialoglycan ligands for Siglec-7 correlate with bone metastasis and reduced survival times in prostate cancer patients [155]. Further studies investigating the specific role of the Siglec-sialoglycan axis in the growth of prostate tumours are ongoing.

## Galectins

Galectin-1 (Gal-1) is the most abundantly expressed Galectin in prostate cancer that is upregulated with progression to CRPC [197]. Gal-1 is highly expressed in CRPC cells but not in androgen-sensitive cells and functions in CRPC growth and invasion through suppression of AR and Akt signalling [198]. Upregulation of Gal-1 has also been linked to AR and AR-V7 expression and their transcriptional activity [199]. Wang et al. recently investigated the role of Gal-1 in the immunosuppressive tumour microenvironment of prostate cancer [200]. This showed Gal-1 is expressed around the tumour stroma and can be secreted by prostate cancer cells. Tumour secreted Gal-1 can bind to receptors on immune cells to induce T cell apoptosis [201]. In line with this, conditioned medium from Gal-1 expressing prostate cancer cells can trigger the apoptosis of T cells and Gal-1 knockdown in prostate cancer cells prevents T cell apoptosis in the tumour microenvironment [200]. Galectin-3 (Gal-3) was initially reported to be decreased in prostate tumours [202]. However, it has since been determined that cleaved Gal-3, rather than intact Gal-3 (detected by older studies), is upregulated in prostate tumours and correlates with tumour progression, metastasis, and PSA levels [203, 204]. Gal-3 is detected in the blood of prostate cancer patients [205, 206] and is implicated as a secreted factor that can alter the tumour microenvironment [189]. In metastatic prostate cancer, Gal-3 can suppress osteoclast differentiation and is crucial to bone remodelling [207, 208]. Gal-3 is also required for prostate tumour cells to establish and maintain immune tolerance through the dysregulation of CD8+ T cell cytotoxic responses [204].

In addition to Gal-1 and Gal-3, other galectins have also been investigated in prostate cancer, with Gal-4, Gal-9 and Gal-12 reported as downregulated in more advanced prostate cancer [197], while Gal-8 is stably expressed during disease progression [209]. Gal-4 can interact with C1GALT1-dependent *O*-glycans and is linked to castration resistance and poor patient prognosis [166, 210] and Gal-8 has been associated with prostate cancer metastasis through modulation of E-cadherin expression and rearrangement of the cytoskeleton [209]. The potential role of other galectins in prostate cancer biology is unknown, but emerging findings from other cancer types functionally implicate Galectins in all of the cancer hallmarks and suggest this is an exciting area to explore [211, 212]. Interestingly, *N*-glycan MSI of prostate tumours has indicated that most tetra-antennary glycans (which are upregulated in CRPC) are non-sialylated, which could be a mechanism leading to increased binding of galectins to prostate cancer cells [35].

## O-GlcNAcylation

In addition to the glycosylation of membrane and secreted proteins, intracellular proteins can also be glycosylated by the addition of *O*-GlcNAc (known as *O*-GlcNAcylation) [213]. This modification occurs on serine or threonine residues on target proteins and plays a fundamental role in numerous cellular processes [213]. *O*-GlcNAcylation is a reversible modification, facilitated by the *O*-GlcNAc transferase (OGT) enzyme and removed by *O*-GlcNAcase (OGA). The hexosamine biosynthetic pathway (HBP) produces UDP-GlcNAc, which is used as a substrate by OGT and plays a key role in the metabolic rewiring observed in cancer [214]. In prostate cancer, *O*-GlcNAcylation is elevated in primary tumours relative to benign disease [215] and levels of *O*-GlcNAc correlate with higher Gleason scores and poor patient prognosis [216]. The OGT enzyme is upregulated in aggressive primary prostate cancer and can regulate the stability of c-Myc [217]. UAP1 (UDP-N-Acetylglucosamine Pyrophosphorylase 1), which is the last enzyme in the HBP pathway, is also upregulated in prostate cancer tissues [218]. Conversely, although HBP is elevated in primary prostate cancer and linked to disease progression, in CRPC the pathway is downregulated and HBP inhibition in CRPC may promote tumour growth [219], suggesting that the requirement of HBP by prostate cancer cells is likely tumour stage dependent and that targeting this pathway therapeutically would need to be carefully managed [220].

## Targeting aberrant glycosylation for prostate cancer therapy

**Fig. 4:**
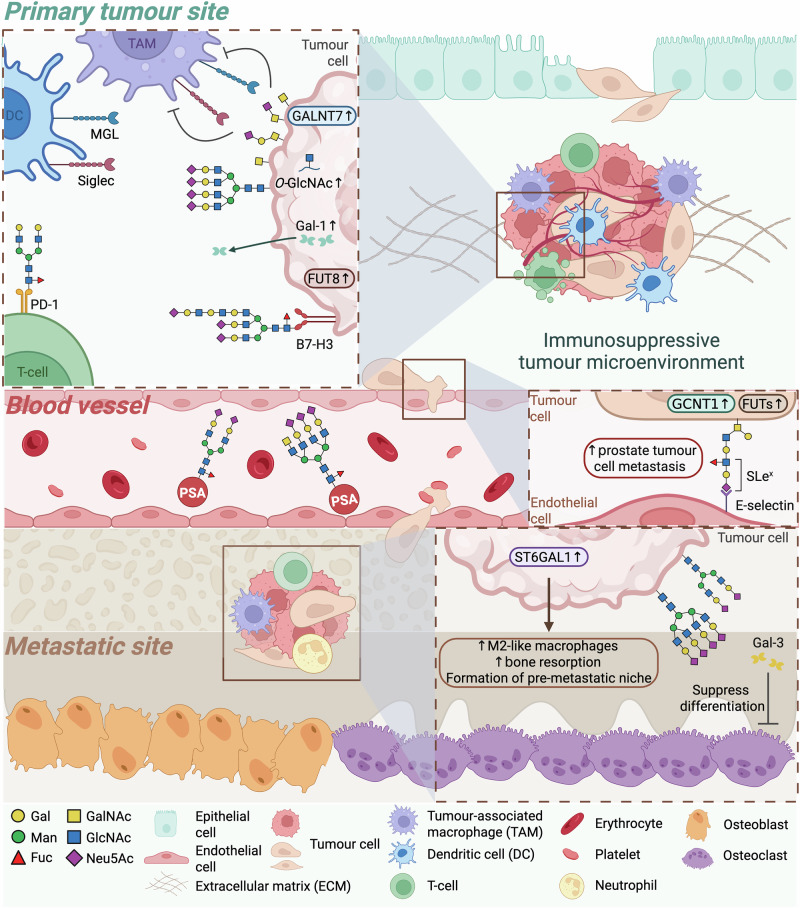
Glycosylation changes in prostate cancer and their role in disease progression. Aberrant glycosylation is linked to multiple oncogenic pathways important in prostate tumour growth, metastasis and immune evasion.

**Fig. 5:**
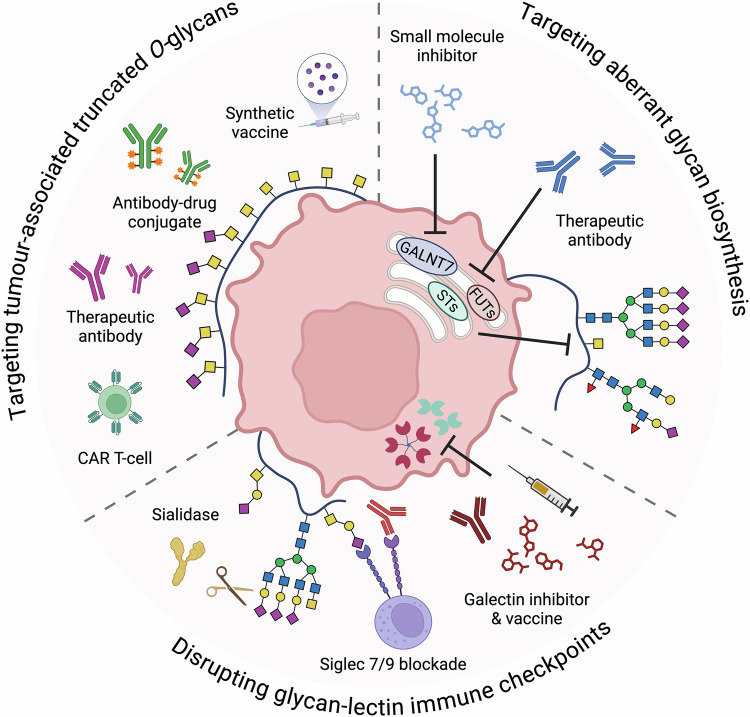
Therapies to target aberrant glycosylation to treat prostate cancer. Potential therapeutic strategies being investigated for prostate cancer therapy include targeting aberrant fucosylation and sialylation, blocking the truncated *O-*glycans Tn and sTn, the use of small molecule inhibitors to target specific glycosyltransferases and strategies to block Galectins and Siglecs.

The targeting of cancer-associated glycosylation is an exciting area of innovation in the development of novel cancer therapies [10, 221]. As discussed in this review, aberrant glycosylation is common in prostate cancer and is linked to multiple oncogenic pathways important in disease progression (Fig. 4). Potential therapeutic strategies being investigated to target aberrant glycosylation to treat prostate cancer include targeting aberrant fucosylation and sialylation, blocking the truncated *O-*glycans Tn and sTn, the use of small molecule inhibitors to target specific glycosyltransferases and strategies to block Galectins and Siglecs (Fig. 5). Targeting fucosylation represents a promising therapeutic strategy and a repertoire of fucosyltransferase inhibitors has been developed that inhibit fucosylated glycans [42]. Fucosylation inhibitor 2-fluorofucose (SGN-2FF) is an orally bioavailable cell-permeable inhibitor that has shown promising anti-cancer effects both in vitro and in vivo, including for prostate cancer [74] and shows direct and indirect effects on cancer cells, immune cells and the tumour microenvironment [222]. Although a clinical trial evaluating the fucosylation inhibitor SGN-2FF demonstrated preliminary evidence of anti-tumour activity, investigations were terminated due to adverse effects (NCT02952989) [223]. However, additional fucosylation inhibitors have now been developed, including the SGN-2FF derivatives A2FF1P and B2FF1P and the metabolic inhibitors Fucotrim I and Fucotrim II [224–226]. These compounds can effectively shut down the synthesis of fucosylated glycans in prostate cancer cells to suppress proliferation and are being developed as new treatments for prostate cancer, although modifications will be needed to render them more targeted toward cancer cells [47].

The targeting of aberrant sialylation is also being explored as a therapeutic strategy for prostate cancer. The sialyltransferase inhibitor P-3F_AX_-Neu5Ac can inhibit the growth of prostate cancer cells [86] and although targeted or localised delivery of this inhibitor can inhibit metastasis and promote T cell immunity in other cancer types [227, 228], at effective doses, this compound is known to induce renal toxicity [229]. Novel C-5-modified 3-fluoro sialic acid sialyltransferase inhibitors are now available, where the natural N-acetamide group is replaced with a carbamate functionality, which are more efficiently metabolised and reach higher effective concentrations within the cell [230, 231]. The therapeutic potential of the C-5 carbamate sialyltransferase inhibitor P-SiaFNEtoc has been investigated in the context of prostate cancer progression. Sialic acid blockade using P-SiaFNEtoc decreases prostate cancer cell growth and downregulates the expression of genes and proteins important in the trajectory of disease progression [47]. Furthermore, the pre-treatment of prostate cancer cells with P-SiaFNEtoc (to remove sialylated glycans) suppresses the growth of prostate tumours and inhibits the metastatic spread of prostate cancer to bone [90]. Together, these studies provide proof-of-concept data to support targeting aberrant sialylation to treat advanced prostate cancer and highlight the potential to further develop this strategy (once safer, more targeted sialylation inhibitors are available) to develop new therapies for advanced prostate cancer.

The dysregulation of glycosyltransferases is recognised as a primary driver of aberrant glycosylation in cancer that can likely be therapeutically exploited [232]. In prostate cancer, proof-of-principle studies highlight glycosyltransferases, including GALNT7, ST3GAL1, ST6GAL1 and FUT8, as attractive targets for prostate cancer therapy. Small molecule inhibitors of these glycosyltransferases, which are directly focused on enzyme inhibition, are being actively developed [233]. Research to develop small molecule inhibitors of GALNT7 is underway [234] and demonstrating the feasibility of this strategy, an isozyme-selective inhibitor has already been discovered for GALNT3 [235]. Selective inhibitors of FUT8 are available, including FDW028, which has demonstrated in vivo efficacy to suppress tumour growth and prolong the survival of mice with metastatic colorectal cancer [77, 236] and a GDP-dependent covalent inhibitor of FUT8 that functions in cells without mimicking the donor substrate [237]. Studies are ongoing to develop and validate specific novel inhibitors of the sialyltransferase enzymes and promising compounds have been identified that show activity and selectivity towards ST3GAL1 and ST6GAL1 [238]. Moving forward, future studies will of course be needed to further develop and refine these inhibitors for therapeutic use. However, current data are encouraging and we anticipate that specific glycosyltransferase inhibitors will be relevant for prostate cancer therapy and are candidates for further investigation.

Truncated *O-*glycans, including the Tn and sTn antigens, drive key hallmarks of malignancy and are linked to poor prognosis across multiple tumour types. Both Tn and sTn are usually absent in healthy tissues and this cancer specificity highlights their potential as attractive targets for therapeutic intervention [239]. Studies show that the Tn antigen is expressed by up to 26% of prostate cancers [139] and sTn is detected in up to half of high-grade prostate tumours [150, 151]. Strategies to inhibit Tn and sTn in cancer include monoclonal antibodies, antibody drug conjugates, vaccines and CAR-T cell therapies, with some therapies progressing into pre-clinical development and clinical trials [240, 241]. However, the therapeutic potential of targeting truncated *O-*glycans in the context of prostate cancer has not yet been investigated and larger studies are needed to explore the prognostic implications of Tn and sTn in larger, well- characterised patient cohorts. Moving forward, we anticipate the use of companion diagnostic tests to identify subsets of prostate cancer patients with high levels of Tn and sTn to stratify those more likely to benefit from treatments targeting these truncated *O-*glycans.

The therapeutic benefit of targeting Galectins in the context of prostate cancer is also being explored. LLS30 is a small-molecule inhibitor of Gal-1, which decreases the binding affinity of Gal-1 to its binding partners [200]. LLS30 has demonstrated efficacy to suppress the in vivo growth of CRPC models [198]. Therapeutic inhibition of Gal-1 using LLS30 can potentiate the anti-tumour effect of docetaxel [198], is reported to re-sensitise prostate cancers to enzalutamide therapy [199] and can suppress T cell apoptosis to enhance the efficacy of anti-PD-1 therapy [200]. Together, these findings underscore Gal-1 as a promising therapeutic target in prostate cancer and highlight the potential of Gal-1 inhibitors to treat advanced disease. Therapeutic strategies to block Gal-1 activity include drugs, antibodies and vaccines, with some progress towards potential clinical trials [242, 243], although not yet investigated in the context of prostate cancer. Gal-3 has been identified as a fundamental player in prostate tumour immune escape, which is a crucial inhibitor for the success of immunotherapy in prostate cancer [204]. Furthermore, Gal-3 is downregulated by low doses of docetaxel and prior treatment with low-dose docetaxel has been proposed as a therapeutic strategy to enhance prostate cancer immunotherapy [204]. Gal-3 is recognised as a multi-mode promoter in a broad range of cancers and is currently a hotly pursued therapeutic target with several inhibitors in pre-clinical development and/or demonstrating encouraging results in early phase clinical trials [244]. The discovery of galectin inhibitors offers promise for the future treatment of prostate cancer and could represent a starting point for the development of novel combination therapies. Further studies will be necessary to fully elucidate the interplay between Galectins and prostate tumour immunity and to evaluate the therapeutic benefit of targeting Galectins in the context of prostate cancer progression.

The Siglec-sialoglycan axis is a glyco-immune checkpoint where sialoglycans expressed by cancer cells bind to inhibitory Siglec receptors on immune cells. This mechanism enables tumours to evade immune surveillance (similar to the PD-1/PD-L1 checkpoint) and is a major target for cancer immunotherapy [245]. Siglec-engaging immunosuppressive sialoglycans are upregulated in prostate tumours compared to normal prostate tissues and Siglec-7 ligands are associated with poor patient prognosis [155]. Antibody blockade of Siglec-7/9 inhibits prostate cancer xenograft growth and increases immune cell infiltration in a humanised mouse model [184] and an engineered dual-action sialidase (E-612), that strips Siglec ligands from prostate cancer cells, can suppress tumour growth and prolong the survival times of mice with prostate cancer bone metastasis [155]. Together, these studies demonstrate that the Siglec-sialoglycan axis is clinically actionable to impede prostate cancer progression and opens up new opportunities to explore new immune-based interventions for prostate cancer therapy.

## Emerging directions and technologies for prostate cancer glycomics

We expect that there are three major areas of research that will enhance and expand on the topic areas covered in this review. The first area is better integration of glycomics with multiplexed spatial transcriptomic and proteomic studies of prostate cancer. There are many published studies in the spatial-omic area for prostate cancer, summarised in reviews [246, 247]. These studies have primarily focused on characterising the roles of the immune microenvironment and cancer- associated fibroblasts in immunotherapy resistance, as well as tumour heterogeneity. Outside of identifying transcripts and proteins that are glycoproteins, none of these studies has linked spatial glycomics or different glycan structural features with glycan biosynthetic and processing genes. There are two studies linking prostate cancer spatial lipidomics with spatial proteomics and transcriptomics [248, 249], so integration of glycomics is feasible. A recent large-scale multi-omics study in glioma integrated spatial transcriptomic, MIBI-tof proteomic profiling and spatial *N*-glycan MS imaging to identify prognostic signatures of glioma grade [250]. This study illustrates the contribution of including glycomic data in these types of large-scale spatial-omic workflows, and it is expected that similar glycomic-centric approaches applied to prostate cancers will be just as informative.

A second area is improvements in using new mass spectrometry approaches for direct identification of prostate cancer-associated glycopeptides for *N*- and *O*-linked glycans, illustrated in some already cited studies [131, 132, 251, 252]. A recent study by the Pitteri lab used site-specific mapping of *N*-linked glycopeptides to undertake deep glycosylation profiling of matched tumour and normal prostate tissues from 24 patients undergoing radical prostatectomy [251]. The study identified 1354 unique proteins with associated glycopeptides, with only ∼34% of glycopeptides shared between cancer and non-cancer tissues. Consistently larger numbers of unique glycoproteins, glycopeptides and glycosites were identified within the prostate cancer group, emphasising that glycosylation is increased and aberrant in prostate cancer and that site-specific mapping is required to fully understand glycosylation changes [251]. In recent years, there have also been coordinated improvements in high-resolution mass spectrometer capabilities combined with new glycopeptide database identification software [253–255]. There are also new *O*-glycoprotease workflows to identify *O*-glycopeptides [256, 257], which have not yet been applied to prostate cancer samples. Improved characterisation of chondroitin sulfate proteoglycans associated with prostate cancer is also expected. This glycopeptide level data can also be integrated and extend existing and future spatial transcriptomic and proteomic studies.

A third area is the new field of cell surface glyco-RNA, originally described as small RNAs that have glycans attached via an amine-linkage that is sensitive to peptide *N*-glycosidase digestion [258]. Subsequent studies identified the specific RNA carrier and glycan linkage sites [259] and more recently, *O*-glycan RNA species have been identified [260]. Functionally, glyco-RNAs seem to be important in regulating specific cell surface processes like immune recognition [261] and interactions with heparan sulfate and growth factors [262]. No specific prostate cancer study has yet been published, although research is ongoing as reported in conference abstracts [263, 264]. It is expected that glyco-RNAs will have some functional influence on prostate cancer development and progression.

## Conclusions

Glycans are major components of cells, defining and modulating several key physiological processes in prostate tumour growth, metastasis and immune evasion. The rapid development of new technologies to profile glycans in cells and tissues over the last decade, alongside advances in our understanding of how glycosylation modulates biological functions, has led to an increased understanding of prostate tumour glycobiology that is becoming clinically actionable. Moving forward, the development of glycosylation-based biomarkers and glycan-targeting drugs and their translation into cancer diagnosis and therapy is likely to have a major impact on the early diagnosis, patient stratification and improved treatment of prostate cancer.
